# Clinical and radiographic comparative study to evaluate the efficacy of restoring destroyed primary incisors using two different techniques—A pilot study

**DOI:** 10.1002/cre2.307

**Published:** 2020-07-14

**Authors:** Seba Ibrahim, Abdul Wahab Nourallah

**Affiliations:** ^1^ Department of Paediatric Dentistry, Faculty of Dentistry Tishreen University Syria

**Keywords:** composite resin post, destroyed primary incisors, early childhood caries, glass fiber post

## Abstract

**Objective:** The restoration of destroyed maxillary primary incisors is difficult because of the insufficient amount of coronal structure. This pilot study aimed to compare the efficacy of composite posts technique and glass fiber posts technique in restoring destroyed primary incisors.

**Materials and Methods:** Thirty‐six destroyed maxillary primary incisors in 11 children with early childhood caries were randomly assigned after endodontic treatment into two groups: glass fiber posts (*n* = 18) and composite resin posts (*n* = 18). Blinded clinical evaluation was made at 3, 6, 9, and 12 months and Blinded radiographic evaluation was made at 6 and 12 months. The evaluation was according to the FDI criteria. Data were analyzed with Fisher's exact test (*α* = .05).

**Result:** After 12 months post‐treatment, the success rates were 88.2% in glass fiber posts group and 70.6% in composite resin posts group. There was no statistically significant difference between the two groups according to the evaluation criteria (*p*‐value >.05).

**Conclusion:** Glass fiber posts technique and Composite posts technique may be used in the restoration of destroyed primary incisors.

## 1 INTRODUCTION

Early childhood caries (ECC) is the most common reason for the destruction of maxillary primary incisors (Ripa, 1988). According to the AAPD (American Academy of Paediatric Dentistry), ECC is the presence of one or more decayed, missing, or filled tooth surface in a 71 months old child or younger (American Academy of Pediatric Dentistry, 2016). ECC proceeds rapidly leading in its extreme cases to loss the full coronal structure (O'sullIvan & Tinanoff, 1993). The extraction of the maxillary primary incisors was the only treatment option when the coronal structure is lost (Carranza & Garcia‐Godoy, 1999). Early loss of these teeth may lead to problems in the aesthetics, self‐esteem, masticatory function, speech, and the development of parafunctional oral habits (Holan & Needleman, 2014). Many parents prefer the restoration of destroyed primary incisors in their children instead of the extraction (Carranza & Garcia‐Godoy, 1999; Holan, Rahme, & Ram, 2009), but this procedure is hard to be done since there is an insufficient amount of coronal tooth structure (Waggoner, 2002). However, endodontic treatment and the application of intracanal reinforcements might be necessary before building the crown in primary incisors with extensive coronal destruction to increase the bonding surface area, so that increases the restoration resistance to masticatory forces (Eshghi, Kowsari‐Isfahan, & Khoroushi, 2013). Cohen et al. (1997) stated that the intracanal post is necessary when there is insufficient or no amount of coronal tooth structure (Sharaf, 2002). However, it must be considered not to interfere with the eruption of the permanent successors (Babaji, Chaurasia, Chaurasia, Masamatti, & Vikram Shetty, 2015; Sharaf, 2002). That requires to apply the reinforcement in the coronal third of the root canal 3 mm under the cemento‐enamel junction (CEJ; Babaji et al., 2015; Sharaf, 2002). Many techniques had been suggested to be used in restoring the destroyed maxillary primary incisors, including the use of intracanal reinforcements such as: composite resin posts (Mendes, Benedetto, Zardetto, Wanderley, & Corra, 2004), composite resin posts with undercut (Judd, Kenny, Johnston, & Yacobi, 1990), omega‐shaped orthodontic wire with flowable composite resin (Mortada & King, 2004), polyethylene ribbon fiber posts (Viera & Ribeiro, 2001), biologic posts (Grewal & Seth, 2008), glass fiber posts (Sharaf, 2002), reversed prefabricated metal posts (Eshghi, Esfahan, & Khoroushi, 2011).

Glass fiber post technique had been used by many researchers in restoring destroyed primary incisors and it showed high success rates (Eshghi et al., 2013; Memarpour, Fereshteh, & Abbaszadeh, 2013; Sharaf, 2002; Subramaniam, Babu, & Sunny, 2008). In this technique, the glass fiber post is lute with either resin cement or flowable composite. The combination between the flowable composite and the glass fiber post forms a firmly attached restoration unit (Sharaf, 2002). The glass fiber post has good aesthetics and mechanical properties close to that in dentin (Lamichhane, Chun, & Zhang, 2014).The disadvantage of this technique is the high cost of fiber posts (Babaji et al., 2015).

Composite post technique is a simple technique to restore destroyed primary incisors. The composite is applied in several layers (2 mm thickness for each layer) into the root canal (Mendes et al., 2004). It had been reported by Grosso et al. (1987; Judd et al., 1990). The shape of the post was tapered, no data were found on the number of treated teeth or failures (Judd et al., 1990). This technique had been reported too in a case report by Mendes et al. (2004). They stated that this technique improved the aesthetics and reduced chair time and cost since one material was used for restoration (Mendes et al., 2004).

There are no previous clinical studies that evaluated the efficacy of nanohybrid composite resin posts in restoring destroyed primary incisors.

This study aimed to evaluate the efficacy of composite posts technique in restoring destroyed primary incisors compared to glass fiber posts technique.

## 2 MATERIALS AND METHODS

**FIGURE 1:**
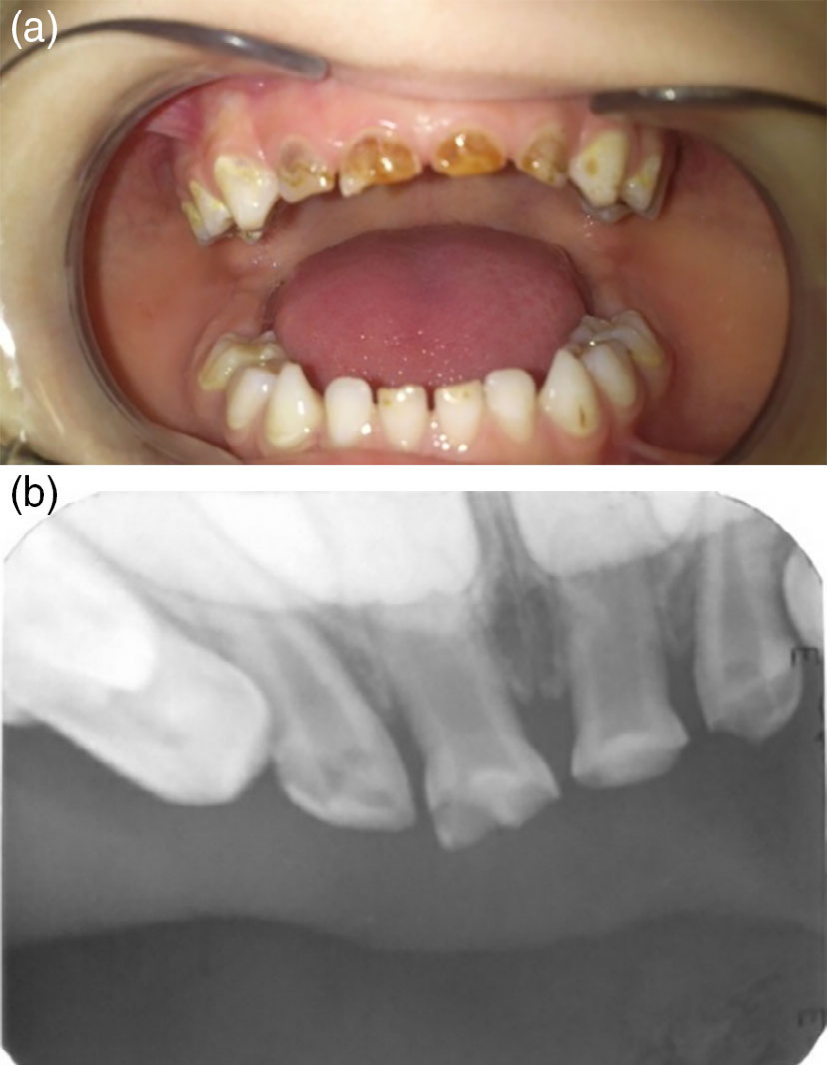
(a) Clinical photograph at baseline. (b) Periapical radiograph at baseline

The study was carried out at the Department of Paediatric Dentistry, Faculty of Dentistry, Tishreen University between November 2017 and August 2018. The protocol of the study had been approved by the Institutional Review Board of Tishreen University under approval (No. 2698) during the session (No. 15). Thirty‐six maxillary primary incisors in 11 patients aged from 2 to 5 years old presenting with ECC were treated in this double‐blind (patient and evaluator) randomized clinical trial. A brief history was recorded, clinical and radiographic examination to the primary teeth was done and clinical photographs (Figure 1a) and periapical radiographs (Figure 1b) to the restorations were taken. A written consent was signed by the parents of the participants after explaining the study protocol to them. The inclusion criteria were: Healthy children (medically and mentally), no malocclusion, no deleterious oral habits, destroyed primary maxillary incisors due to ECC (the destruction involved three fourths of the crown structure), sufficient amount of the root with no more than one third of the root physiologic external resorption compared with the adjacent teeth, no internal root resorption. The exclusion criteria were: mobility of the teeth and subgingival crown destruction.

The maxillary primary incisors were randomly assigned to receive either glass fiber post restorations or composite post restorations. The restorative techniques were selected consecutively from the first to the last tooth, and the first technique to the first tooth had been chosen by throwing a coin. At least two maxillary primary incisors in each patient's mouth were treated with the two techniques. All teeth were treated by one well‐experienced operator.

Local anesthesia was made to the teeth and isolation was made with cotton rolls and a high‐volume saliva ejector. Caries was removed using a (no 14) round diamond bur (DIAMANT®, HORICO, Germany), and the pulp tissue was extirpated. Working length was determined 2–3 mm shorter than the radiographic length, then the root canals were prepared using endodontic files (Nos. 30, 35, 40; K‐file, Radix, Czech Republic) under irrigation with sodium hypochlorite (3%), saline, and then chlorhexidine (0.12%; Oralfresh K®, Biofresh, Syria). The canals were dried with paper points and obturated with zinc oxide and eugenol past (ZOE).

The coronal third of the root canal was emptied from the ZOE past 4 mm under the CEJ using fisher diamond bur (No. 10; DIAMANT®, HORICO, Germany) and a 1 mm layer of light‐cured glass ionomer cement (Fusion i‐Seal™, PREVEST DentPro., India) was applied above the ZOE to isolate the resin restoration material, so that a 3 mm length of the canal was left as a post space.

**FIGURE 2:**
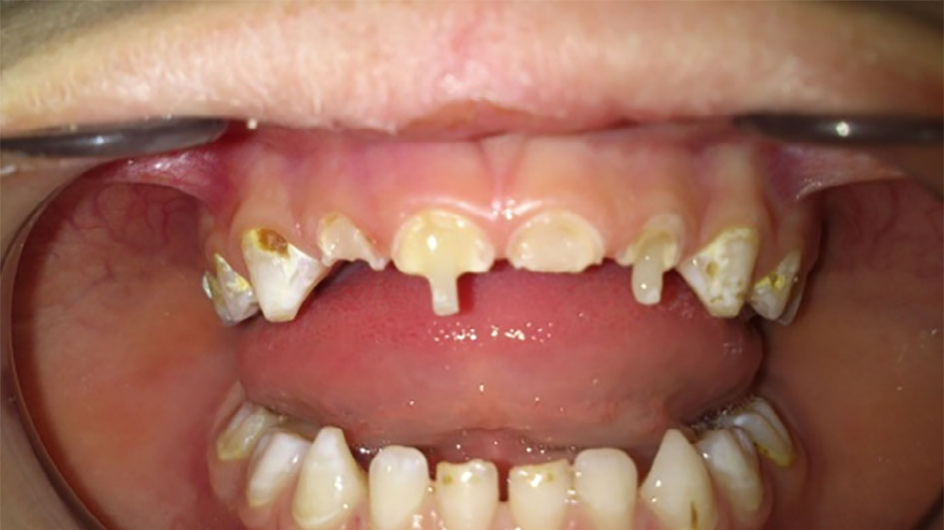
The posts in the canals

In group A (glass fiber post): The post (Bioloren®, Italy) with proper size was cut to obtain the length of 6 mm and wiped with ethanol according to manufacturer's instructions. The tooth surfaces were etched with phosphoric acid 37% (Condac® 37, FGM, Brazil) for 30 s for enamel and 15 s for dentin, then rinsed with water and dried with air. A two‐step etch‐and‐rinse adhesive system (Tetric N‐Bond®, Ivoclar Vivadent, Liechtenstein) was applied and thinned with the weak air stream and light‐cured for 20 s according to manufacturer's instructions with a light‐curing unit (BLUEDENT® LED smart, BG LIGHT LTD, Germany). Light‐cured flowable composite resin (Te‐ Econom Flow®, Ivoclar Vivadent, Liechtenstein) shade A2 was injected into the canal and the post was inserted and fitted by gentle finger pressure, then the complex was light‐cured with the same curing unit for 120 s (Figure 2). The core was built with flowable composite and the crown was built with light‐cured nanohybrid composite resin (Tetric N‐Ceram®, Ivoclar Vivadent, Liechtenstein) using appropriate celluloid crown.

In Group B (composite post): The 3 mm coronal third of the root canal was etched with phosphoric acid 37% (Condac® 37, FGM, Brazil) for 15 s and also the other tooth surfaces (30 s for enamel and 15 s for dentin) then rinsed and dried. The same adhesive system was applied according to manufacturer's instructions. The light‐cured nanohybrid composite resin shade A2 was applied into the root canal in several layers (2 mm thickness for each layer) and each layer was light‐cured with the same light‐curing unit (Figure 2). The crown was built with the same light‐cured composite resin using appropriate celluloid crown.

**FIGURE 3:**
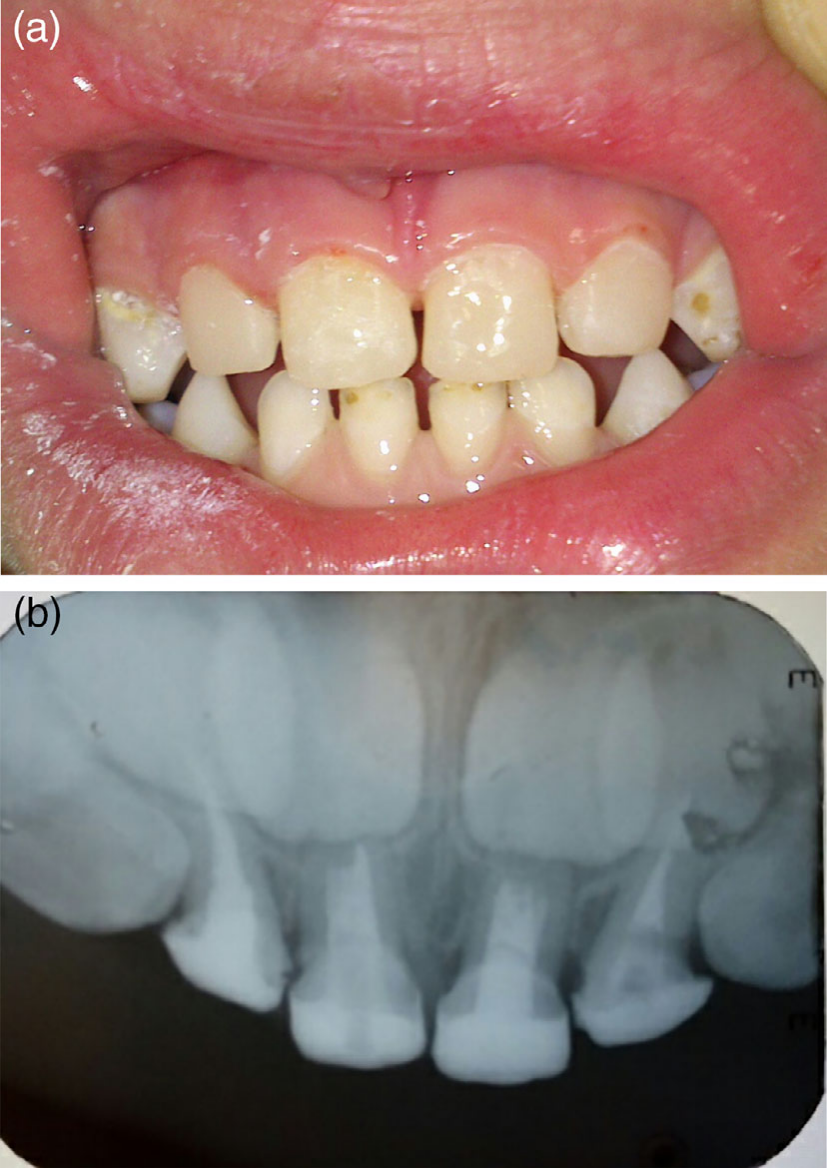
(a) The final restorations at baseline (clinical). (b) The final restorations at baseline (radiographic)

In both groups, occlusion was checked. Finishing and polishing were performed using fin diamond burs and flexible aluminum oxide disks. Finally, clinical photographs (Figure 3a) and periapical radiographs (Figure 3b) to the restorations were taken.

**FIGURE 4:**
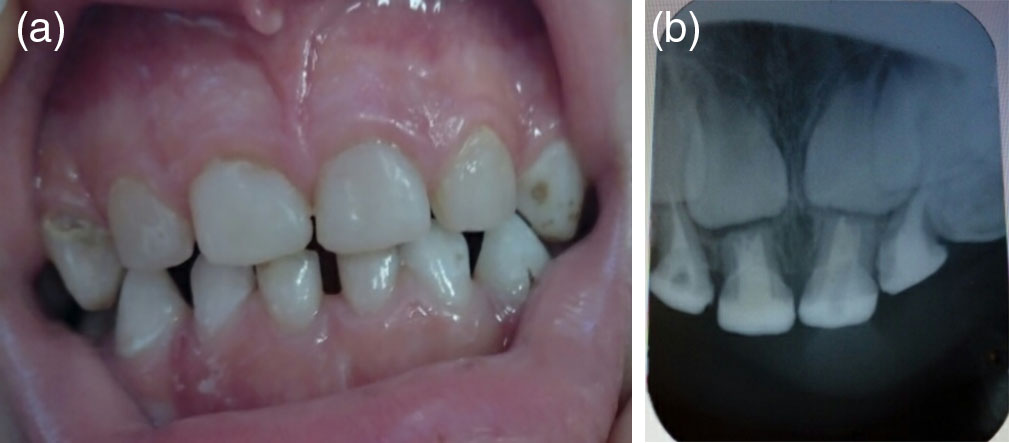
(a) At the 12‐months follow‐up appointment. (b) At the 12‐months follow‐up appointment

The restorations were followed‐up clinically after 3, 6, 9, and 12 months (Figure 4a) and radiographically after 6 and 12 months (Figure 4b) from the baseline. Clinical examination was made using visual inspection and dental explorer at the follow‐up appointments by the operator and clinical photographs were taken to be assessed by an independent evaluator. Periapical radiographs were taken at the radiographic appointments to be assessed by another independent evaluator for radiographic evaluation. The evaluation was according to FDI criteria (Hickel et al., 2007). The outcome was considered to be successful if the following: The post and the crown were in situ with no dislodgement or debonding or movement of one of them (clinically), the absence of clinical or radiographic evidence of secondary caries, no root and/or post fracture (radiographically), no marginal discoloration that reaches to the dento‐enamel junction, intact marginal integrity without catching of the explorer or visible crevice reaches to the dento‐enamel junction, the absence of clinical or radiographic signs of endodontic or periodontic conditions requiring endodontic retreatment or extraction of the tooth.

### 2.1 Statistical analysis

Statistical Analysis was done using IBM SPSS Statistics (version 20) by the operator. Statistical significance of difference was tested using Chi‐square test for independence of attributes if cell frequency is larger than 5; else Fisher's exact probability test was applied. The differences were considered significant when *p*‐value ≤.05.

## 3 THE RESULTS

Eleven children (six girls and five boys) aged 2–5 years with 36 destroyed primary maxillary incisors were involved in this study. Each group from the two groups had 18 incisors, in Group A (glass fiber post) there were 11 central incisors and 7 laterals, and in Group B (composite post) there were 10 central incisors and 8 laterals.

**FIGURE 5:**
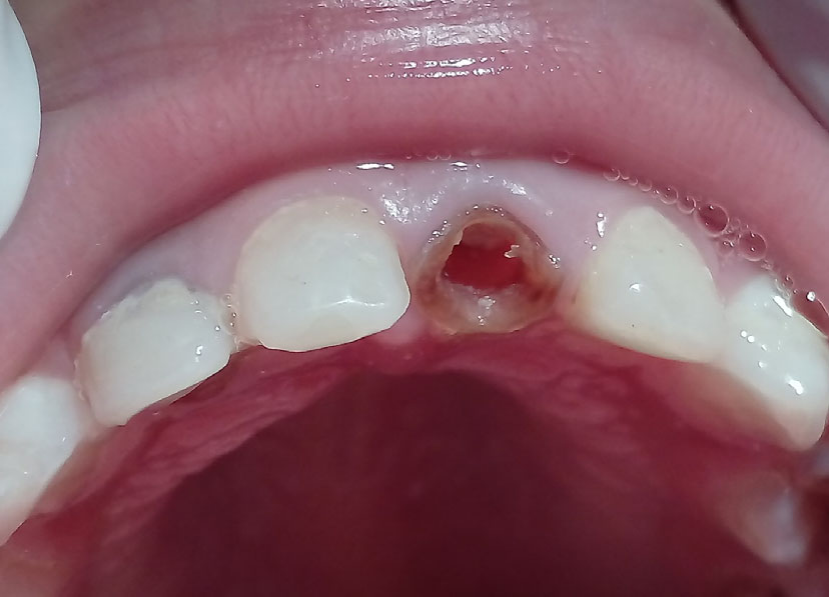
Post debonding from the root canal with complete loss of the restoration in tooth number 61 at the 3‐months post‐treatment

**FIGURE 6:**
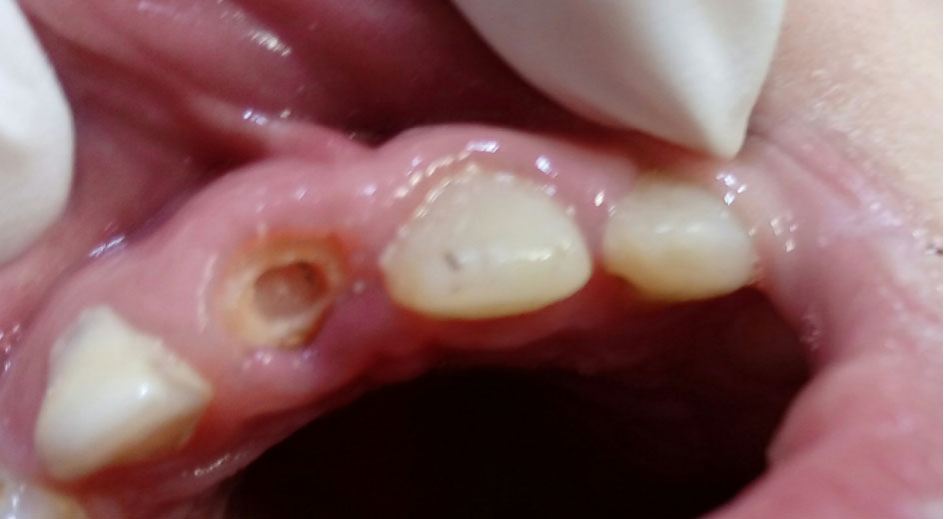
Post complete fracture at the level of canal orifice with complete loss of the restoration in tooth number 51 at the 3‐months post‐treatment

At the 3‐month follow‐up appointment, three complete failures in three different children had been recorded in composite post group. The failure types were: post debonding from the root canal with complete loss of the restoration (Figure 5) in two cases, and post complete fracture at the level of canal orifice with complete loss of the restoration in another case (Figure 6).

**FIGURE 7:**
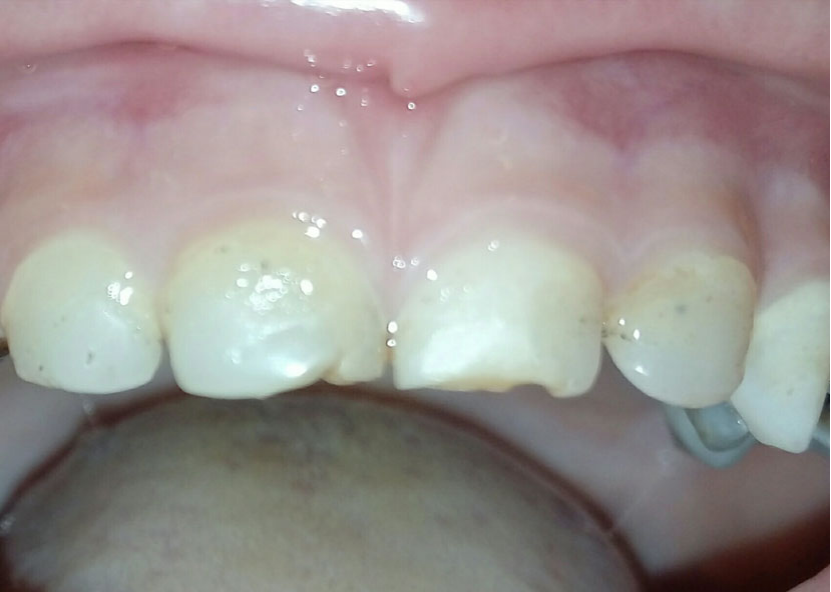
Formation of visible crevice reached to the dento‐enamel junction in tooth number 51 at the 6‐months post‐treatment

At the 6‐month follow‐up appointment, two complete failures in two different children had been recorded in glass fiber post group. The failure types were: post debonding from the root canal with complete loss of the restoration in one case, and the formation of visible crevice reached to the dento‐enamel junction in another case (Figure 7).

At the 9‐month follow‐up appointment, one child with one restoration in each group had been dropped out because of moving out of the city. Two complete failures in two different children had been recorded in composite post group, and the failure type was post debonding from the root canal with complete loss of the restoration.

No failures had been recorded in any of the two groups at the 12‐month follow‐up appointment.

In all of the follow‐up appointments, no root fracture, secondary caries, or periodontic conditions requiring endodontic retreatment had been recorded.

The success rates of the two groups are shown in Table 1. The differences between the two groups at all the follow‐up appointments were statistically insignificant (*p*‐value >.05).

**TABLE 1:** The success rates of the two groups at the follow‐up appointments

Follow‐up appointment	Success rates				*p*‐Value
	Group A (glass fiber post)		Group B (composite post)		
	*n* (%)	*N*	*n* (%)	*N*	
3 month	18 (100)	18	15 (83.33)	18	.229
6 month	16 (88.89)	18	15 (83.33)	18	.489
9 month	15 (88.2)	17	12 (70.6)	17	.224
12 month	15 (88.2)	17	12 (70.6)	17	.224

*Note:* No significant differences between groups (*p* > .05).

## 4 THE DISCUSSION

In this study, the efficacy of composite post technique was compared with glass fiber post technique in restoring the maxillary primary incisors with extensive carious lesions that affected the full coronal structure. Both restoration techniques were applied in each child's mouth to ensure that both posts were in the same oral environment for comparison.

In both groups, the post was applied in the coronal third of the root canal 3 mm under the CEJ at the level of the bone to prevent the interference with physiologic root resorption and the eruption of permanent successors as it had been recommended by the previous studies (Mendes et al., 2004; Sharaf, 2002). However, a long‐term follow‐up is needed until the physiologic exfoliation of the treated incisors and the eruption of the permanent successors.

The glass fiber post has a modulus of elasticity (54 GPa) close to that in dentin (approximately 18 GPa; Lamichhane et al., 2014). It was lute with flowable composite in Group A (glass fiber post) as described in previous in vivo (Mehra et al., 2016; Sharaf, 2002; Subramaniam et al., 2008; Vafaei et al., 2016) and in vitro (Baghalian, Ranjpour, Hooshmand, Herman, & Ebrahimi, 2014; Memarpour et al., 2013; Sharaf, 2002) studies which had reported high success rates in this technique.

Nanohybrid composite resin that had been used to build the post in Group B (composite post) had a modulus of elasticity (11 GPa) according to manufacturer's scientific documentation (Tetric N‐Collection, 2010) and it is close to that in dentin. No undercut was made in the post space in Group B because the removal of deep dentin might weaken of the root (Babaji et al., 2015). Besides, no alpha or gamma shaped orthodontic wires were applied because making it is hard to be done and takes extra time (Babaji et al., 2015).

In the present study, post debonding from the root canal walls with complete loss of the restoration had happened in one case in Group A (glass fiber post) because of dental trauma, and four cases in Group B (composite post), one case because of dental trauma, two cases had happened while eating, and one failure case had no specific reason. However, the polymerization shrinkage of the composite material or inadequate isolation might be a potential reason for the failure.

Post fracture with complete loss of the restoration had been recorded in one case in Group B at the 3‐month follow‐up appointment, and it had happened at the level of canal orifice because of dental trauma.

The visible crevice reached to the dento‐enamel junction that had been recorded at the 6‐months follow‐up appointment in Group A happened because of a fracture in the restoration material with the tooth structure that had connected to it.

In the present study, the success rates after 12 months post‐treatment were 88.2% in Group A and 70.6% in Group B, the difference between the two groups was statistically insignificant.

In a study by Judd et al. (1990), the success rate was 100% in the primary incisors restored with composite posts. They referred to the undercut that had been made in the post space as the reason for the success (Judd et al., 1990).

Sharaf (2002) stated that the glass fiber posts that had been lute with flowable composite resin and used to restore 30 maxillary incisors were fully intact after 12 months post‐treatment. The success rate in that study was 100% (Sharaf, 2002). The higher success rate from that in the present study might be because the reason for failures mentioned above in the glass fiber post group in the present study did not happen in Sharaf's study.

Eshghi et al. (2013) reported a 84% success rate in glass fiber post group after 12 months post‐treatment. This result is close to our result in glass fiber post group in the present study, note that they used a dual‐cured resin cement for luting the glass fiber posts. On the contrary, flowable composite resin was used for luting the glass fiber posts in the present study. The success rate in composite post group in their study was 98% and it's higher than that in the present study. This might be because of the undercut that had been made in their study in the post space which might increase the retention of the post and prevent post debonding.

Vafaei et al. (2016) reported a 74.4% success rate in glass fiber post group after 12 months post‐treatment (Vafaei et al., 2016). This rate is lower than the success rate of the glass fiber post group in the present study. This might be because they treated destroyed primary canines where the eccentric movement loads were applied. On the contrary, destroyed primary incisors were treated in the present study.

Mehra et al. (2016) reported a 93.33% success rate in glass fiber post group after 12 months post‐treatment. The failures in their study had happened because of biting on hard food. However, the success rate in their study is higher than the success rate in glass fiber post group in the present study. The success rate in the composite post group in that study was 60% they referred this result to the polymerization shrinkage in the flowable composite resin material that had been used alone without orthodontic wires to build the post in their study. This success rate is lower than the success rate in the composite post group in the present study where nanohybrid composite resin which had better physical properties than flowable composite resin was used to build the post.

## 5 CONCLUSION

Based on this study results, glass fiber post technique and composite post technique showed acceptable clinical and radiographic success rates after 12 months post‐treatment. The success rate in glass fiber post group was higher than that in composite post group with a statistically insignificant difference. Both techniques may be used in the restoration of destroyed primary incisors.

*Why this paper is important for pediatric dentists*

- Restoring the destroyed maxillary primary incisors in very young children is very important to protect the aesthetics, articulation, and mastication.
- The intracanal reinforcements increase the retention of the restorations in such cases.
- These reinforcements must be only in the coronal third of the root canal to prevent the interfere with the eruption of the permanent successors.

## CONFLICT OF INTEREST

The authors declare no conflict of interest.

## AUTHOR CONTRIBUTIONS

We declare that all authors have made substantial contributions. Dr. Nourallah and Ms. Ibrahim designed the research study. Ms. Ibrahim performed the research including patient recruitment, data collection, and data analyzing. Ms. Ibrahim prepared the manuscript draft. Dr. Nourallah revised the manuscript. All authors approved the final manuscript. All authors had complete access to the study data. All authors agree to be accountable for all aspects of the work in ensuring that questions related to the accuracy or integrity of any part of the work are appropriately investigated and resolved.
